# Epigenetic Regulators of Mesenchymal Stem/Stromal Cell Lineage Determination

**DOI:** 10.1007/s11914-020-00616-0

**Published:** 2020-08-13

**Authors:** Dimitrios Cakouros, Stan Gronthos

**Affiliations:** 1 Mesenchymal Stem Cell Laboratory, Adelaide Medical School, Faculty of Health and Medical Sciences, University of Adelaide, Adelaide, SA Australia; 2 Precision Medicine Theme, South Australian Health and Medical Research Institute, Adelaide, SA Australia

**Keywords:** Mesenchymal stem/stromal cells, MSC, Epigenetics, Skeletal stem cells, DNA methylation, Histone methylation, Histone acetylation

## Abstract

**Purpose of Review:**

Although many signalling pathways have been discovered to be essential in mesenchymal stem/stromal (MSC) differentiation, it has become increasingly clear in recent years that epigenetic regulation of gene transcription is a vital component of lineage determination, encompassing diet, lifestyle and parental influences on bone, fat and cartilage development.

**Recent Findings:**

This review discusses how specific enzymes that modify histone methylation and acetylation or DNA methylation orchestrate the differentiation programs in lineage determination of MSC and the epigenetic changes that facilitate development of bone related diseases such as osteoporosis. The review also describes how environmental factors such as mechanical loading influence the epigenetic signatures of MSC, and how the use of chemical agents or small peptides can regulate epigenetic drift in MSC populations during ageing and disease.

**Summary:**

Epigenetic regulation of MSC lineage commitment is controlled through changes in enzyme activity, which modifies DNA and histone residues leading to alterations in chromatin structure. The co-ordinated epigenetic regulation of transcriptional activation and repression act to mediate skeletal tissue homeostasis, where deregulation of this process can lead to bone loss during ageing or osteoporosis.

## Introduction

The stromal component within bone marrow is a hierarchical continuum of mature functional stromal populations and committed progenitor cells. This is sustained by a minor population of long-lived, self-renewing and multipotential skeletal stem cells or bone marrow-derived mesenchymal stem/stromal cells (BMSC) [1]. Purified preparation of human BMSC exhibit the potential to form osteoblasts, adipocytes, chondrocytes, smooth muscle cells and myelosupportive fibroblasts under inductive conditions in vitro or when transplanted in vivo [2–4]. It is well known that the master regulatory transcription factors Runt-related transcription factor 2 (RUNX2), peroxisome proliferator-activated receptor gamma 2 (PPARγ2), myogenic differentiation (MOYD) and sex-determining region Y-box 9 (SOX9) are critical mediators of BMSC differentiation towards the osteogenic, adipogenic, myogenic and chondrogenic lineages, respectively [5–7]. The co-ordinated expression of RUNX2, PPARγ2, MYOD or SOX9 depends on the regulation of chromatin, allowing the activation of these genes and their targets. Therefore, it is critically important to understand the epigenetic modifications regulating BMSC cell fate and lineage-specification.

Epigenetics is the cellular modification of reversible and heritable changes in gene expression that occur without changes in the DNA code [8]. Epigenetic modifications such as DNA methylation and histone modifications regulate the structure of chromatin, which determines the accessibility of genes to transcription factors and other modulators involved in gene regulation. Chromatin is formed through the compaction of DNA strands wrapped around nucleosomes [9], which are linked by histone proteins. The functional state of chromatin, whether open or compact, can be modified through methylation of DNA at cytosines within CpG dinucleotides and on amino acid residues within the histone proteins. DNA methylation is often associated with gene silencing, whereas histone modifications can be mediators of either gene activation or repression. This review will summarise our current understanding of the association and role of epigenetic modifications in BMSC lineage commitment.

## Enzymes Regulating Histone Methylation/Acetylation in BMSC Differentiation

**Fig. 1:**
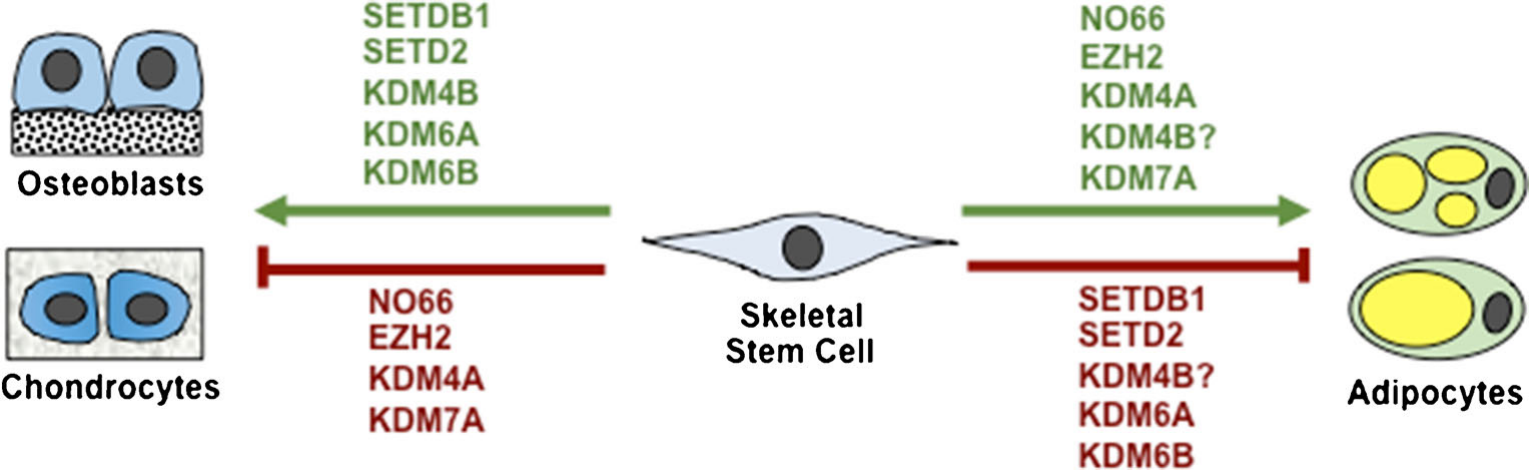
Epigenetic switches regulating skeletal stem cell fate determination. Known enzymes mediating skeletal stem cell osteogenic, chondrogenic or adipogenic commitment. (?) Conflicting functions

The histone 3 lysine 27 (H3K27) methyltransferase, EZH2, is essential for embryogenesis and postnatal tissue development [10–12]. We have recently reported an essential function of EZH2 as an epigenetic switch mediating the function of human BMSC. In human BMSC, EZH2 is a promoter of adipogenesis and inhibitor of osteogenesis via tri-methylation of H3K27 (H3K27me3) of Runx2 and osteopontin genes [13••]. In contrast, the H3K27me3 demethylase, KDM6A, was found to inhibit BMSC adipogenesis and promote osteo/chondrogenesis [13••]. Similarly, another H3K27me3 demethylase, KDM6B, was also discovered to be essential in osteogenic differentiation of MSC [14–17]. We and others have recently expanded these studies in conditional knockout mice where EZH2 is specifically deleted in BMSC leading to increased bone formation with disrupted bone patterning, increased trabecular bone, and increase bone marrow fat [18, 19]. The role of EZH2 in murine adipogenesis acts by binding to β-catenin associated genes, Wnt1, 6, 10a, 10b, 2b, 3a, 8a, 2 and 11 in peripheral pre-adipocytes and their repression promotes adipogenesis [20]. In a mouse model of osteoporosis induced by oestrogen deficiency following bilateral ovariectomy, EZH2 protein and RNA levels were found to be elevated in BMSC isolated from osteoporotic bone samples, with EZH2 and H3K27me3 being highly enriched on promoters of Wnt1, Wnt6 and Wnt10a leading to repression of these bone promoting genes [21] and promoting adipogenesis. Therefore, EZH2/KDM6A/B represent a novel epigenetic switch for controlling BMSC cell fate decisions, part of a complex circuitry network of epigenetic regulators (Fig. 1).

Methylation of histone 3 on lysine 4 (H3K4me3) is associated with transcriptional activation and pivotal for differentiation [22, 23]. Absent, small or homeotic disc1 like (Ash1l) is a member of the Trx family that methylates H3K4. Both Ash1l and H3K4me3 were upregulated in multipotent mouse C3H10T1/2 cells or human BMSC cells undergoing osteogenic, adipogenic or chondrogenic differentiation [24]. Depletion of Ash1l impaired osteo/chondrogenic differentiation but increased adipogenesis, due to a decrease in Hoxa10, Sox9 gene expression and an activation of PPARγ2 expression [24]. Of note, Ash1l and H3K4me3 were found to be downregulated in murine and human osteoporotic bone samples. Ash1l cooperates with other H3K4 methyltransferases such as MLL1 [25, 26], which activate Hox genes [27] involved in osteogenesis. The H3K4 demethylase, RBP2, occupies the osteoblast-specific transcription factor Osterix transcription start site to remove H3K4me3 [28]. Conversely, the H3K4 methyltransferase Set1, part of the COMPASS complex (consisting of MLL, KDM6A, Set1, WDR5), deposits H3K4me3 on the Osterix promoter during osteogenesis [29]. Similarly, the co-factor WDR5 can induce H3K4me3 on the Runx2 promoter, whereas KDM6A removes H3K27me3 during osteogenesis [30]. The balance between competing epigenetic factors is further illustrated by the demethylase, KDM5A, which removes H3K4me3 and inhibits osteogenesis by repressing Runx2 expression [31]. In contrast, KDM7C was shown to promote osteogenesis by enhancing Runx2 binding to osteogenic promoters via demethylation of the Runx2 gene [32].

Another important epigenetic modification is methylation of histone 3 lysine 9 (H3K9me), a marker for gene repression. The H3K9me demethylase, KDM4A, was found to promote adipogenesis and conversely inhibit osteogenesis in primary cultures of BMSC [33]. Subsequent investigations revealed that the Wnt pathway was directly inhibited by KDM4A. However, KDM4A can also act indirectly on bone by reducing DNA methylation on the promoters of the adipogenic associated factors, C/EBPα and SFRP4, leading to activation of adipocyte differentiation. In other investigations, knockout of the H3K9 methyltransferase, SETDB1, was found to cause bone defects in mice [34], implying that SETDB1 is a promoter of osteogenesis. In contrast, knockdown of SETDB1 expression resulted in increased adipogenesis in vitro [35], indicative of another epigenetic mediator of BMSC lineage commitment via H3K9 modification of osteogenic and adipogenic associated gene sets.

Some epigenetic enzymes exhibit different functional roles targeting more than one epigenetic mark such as NO66, a H3K4 and H3K36 demethylase, which has been shown to bind to the Osterix promoter and inhibit transcription [36]. NO66 conditional knockout mice, in the MSC lineage, exhibit increased length and body weight, which is associated increased intramembranous and endochondral ossification [37]. Similarly, the demethylase, KDM7A, was reported to promote adipogenesis but inhibit osteogenesis [38] through demethylation of both H3K9me2 and H3K27me2. KDM7A action resulted in an activation of gene expression levels for the adipogenic associated factors, C/EBPα and SFRP1 a known Wnt inhibitor.

**Fig. 2:**
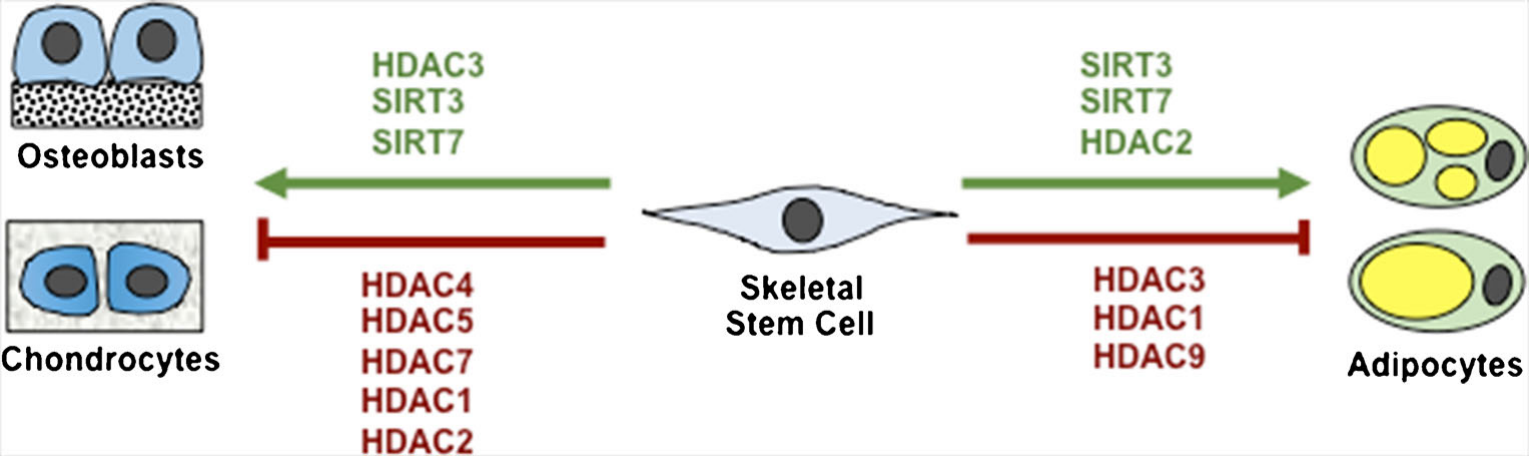
Histone deacetylases regulating skeletal stem cell fate determination. Known histone deacetylase enzymes regulating osteogenesis, adipogenesis and chondrogenesis

Histone acetylation is an epigenetic modification associated with transcriptional activation, where histone deacetylation causes chromatin to compact, leading to transcriptional repression [39, 40]. Members of the histone deacetylases (HDAC), HDAC 3, 4, 5 and 7 are critical for endochondral bone formation [41, 42]. HDAC3 deletion in chondrocytes leads to embryonic lethality, whereas postnatal deletion of HDAC3 delays ossification, growth plate maturation with increased osteoclast activity [43]. The phenotype was associated with increased cytokine and matrix-degrading genes due to increased NF-κB acetylation and decreased bone development genes [43]. An epigenetic library screen identified Abexinostat, which increased H3K9Ac levels on osteogenic/adipogenic genes and promoted both osteogenic and adipogenic differentiation [44••]. This study highlights the utility of screening chemical and small peptide libraries in order to develop new-generation agents to modify histone acetylation patterns controlling MSC cell fate determination (Fig. 2).

Investigations into mechanical bone stimulation demonstrated enhanced Notch signalling and downregulated HDAC1, in human osteoporotic BMSC [45••]. HDAC1 was found to inhibit bone formation and Notch signalling via deacetylating JAG1 and inhibiting HDAC1 attenuated hind limb unloading induced osteoporosis [45••]. Aged BMSC are characterised by reduced differentiation and proliferation, processes which are known to be mediated by HDAC and longevity genes, such as the sirtuins. The mitochondrial deacetylase, Sirt3, was found to be downregulated during culture expansion but was a promoter of both osteogenic and adipogenic differentiation, and attenuated oxidative stress and senescence [46]. Knockout studies of another deacetylase, Sirt7, found that mice developed osteopenia as Sirt7 interacted with Osterix, deacetylating K368, increasing its transactivation and function [47]. A recent study reported that HDAC4 deficient mice display premature ossification due to early-onset chondrocyte hypertrophy [48]. Similarly, HDAC3 deletion specifically in chondrocytes caused delayed angiogenesis, increased bone resorption and severely reduced bone median density [43]. HDAC3 was found to be essential for Collagen type 2 gene expression, while repressing NF-κB and STAT signalling [43].

To date, the gene promoter of the osteogenic master regulatory factor, Runx2, has been reported to undergo specific changes in epigenetic modifications including, enrichment of histone 3 acetylation (H3Ac), H3K4me3 and reduced H3K4me1, H3K27me3 and H4R3me2 [49]. Chromatin immunoprecipitation (ChIP) analysis showed increased binding of WDR5 and KDM6A on the Runx2 promoter during osteogenesis. The enzymes MLL2, MLL3 and Menin, part of the MLL2/MLL3/COMPASS complex, were also found to be present and acted to promote H3K4me3 to activate Runx2 transcription. Amongst the H3K4 demethylases, LSD1, a flavin-containing amino oxidase that removes H3K4me1 and K4me2 was shown to be an inhibitor of osteogenesis in human adipose-derived stem cells. Furthermore, conditional deletion of LSD1 in MSC resulted in a phenotype of short stature, lower body weight, delayed cartilage development and endochondral bone formation, with bones showing increased trabecular and cortical bone volume with increased osteoblast activity. ChIPseq analysis of calvarial cells showed increased H3K4me2 levels with Gene Ontogeny pathway analysis indicating many genes as being involved in bone differentiation including Wnt7b and Bmp2 [50••]. The H3K36me3 mark is a modification orchestrated by SETD2, which is positively correlated with transcription, prevalent on gene bodies and associated with longevity. SETD2 and H3K36me3 levels have been shown to decrease during adipogenesis but increase during osteogenesis [51]. MSC specific conditional knockouts found that SETD2 inhibits adipogenesis and PPARγ1/2, C/EBPα and Fabp4 gene expression and promotes osteogenesis. Moreover, SETD2 knockout mice showed increased marrow fat and reduced bone mass (both trabecular and cortical) in distal femurs indicative of age-associated osteoporosis.

Collectively, these studies demonstrate that the interplay between different epigenetic factors targeting histones is delicately balanced in order to orchestrate correct developmental pathways by targeting lineage-specific gene sets that mediate BMSC cell fate determination.

## Enzymes Regulating DNA Methylation/Hydroxylation in BMSC Differentiation

There is overwhelming evidence implicating DNA methylation as an inhibitor of BMSC osteogenic and adipogenic differentiation in vitro [52••, 53, 54]. DNA methylation has also been shown to affect mechanical bone loading, acting to maintain bone density in vivo, with unloading leading to increased DNA methylation, bone loss and disuse osteoporosis [55, 56]. Biomechanical force has been found to induce important BMSC lineage pathways such as Wnt and Bmp signalling [57, 58] and more recently the Sonic hedgehog pathway (SHH) [59]. The DNA methyltransferase, DNMT3B, is reported to be upregulated at the initial stage of fracture repair and mainly expressed in chondrogenic progenitors, where chondroblast specific deletion of DNMT3B results in diminished fracture repair. Whilst the promoter of SHH was found to be hypermethylated, methylation levels decreased following mechanical stimulation. Moreover, DNMT3B was discovered to methylate the SHH gene promoter, and was dissociated from the promoter following mechanical stimulation, leading to a reduction of methylation levels and gene activation [59].

In more recent years, the hydroxylases, Ten-eleven-translocases (Tet1, Tet2 and Tet3) have emerged as key regulators of stem cell renewal. They catalyse the conversion of 5-methylcytosine (5mC) into 5-hydroxymethylcytosine (5hmC) where it opposes the function of 5mC and leads to DNA demethylation [60–62]. In human BMSC, Tet1 was found to be repressed during osteogenesis and adipogenesis, whereas Tet2 was found to promote both osteogenesis and adipogenesis [52••]. It should be noted that both Tet1 and Tet2 were found to be downregulated in human and murine BMSC derived from osteoporotic bone samples, correlating to reduced 5hmC levels in situ [52••, 63••]. Mechanistically, Tet2 was found to hydroxymethylate Runx2 and Bmp2, resulting in activation of transcription. In other studies, however, both Tet1 and Tet2 were found to promote both osteogenesis and chondrogenesis in murine mesenchymal cells [64] and murine BMSC. This occurred via demethylation of P2rX7, which was found to act as an activator of Runx2 gene expression [63••]. In vivo analyses showed deletion of both Tet1 and Tet2 resulted in impaired self-renewal of murine BMSC, and was associated with a severe osteopenic phenotype. Studies of chondrogenesis revealed that global 5hmC levels increased over time correlating to increased expression levels of Tet1, Tet2 and Tet3 [65]. Knockdown of Tet1 had the greatest effect on 5hmC levels. Highest levels of 5hmC were observed in gene bodies and in promoters preceding transcription start sites on chondrogenic genes such as Sox5, Sox6 and Sox9. Meta-analysis of genes acquiring 5hmC identified pathways essential to cartilage development such as the WNT pathway and combining expression data with 5hmC data showed a close correlation between 5hmC and transcription.

The complexity of BMSC cell fate determination is dependent on interacting epigenetic modifications that control key lineage-specific transcription factors. Osterix is one of the most important lineage determinants for bone formation and is expressed specifically by pre-osteoblasts and chondrocytes [1, 66]. Examination of epigenetic modifications on the Osterix promoter showed that MSC differentiation into osteoblasts, correlated to enriched levels of H3Ac/H3K4me3 accompanied by a decrease in H3K9me3/H3K27me3 [29]. In non-mesenchymal cells, the Osterix promoter was found to be enriched in SUV39H1 and EZH2, which deposit H3K9me3 and H3K27me3 modifications, respectively. Moreover, the analysis of MSC identified several epigenetic modifiers, RNAPII, HDAC1/2/4, Setdb1, JMJD2a, EZH2, JMJD3 and KDA6A bound on the Osterix promoter [29]. Osteogenic differentiation caused enrichment of RNAPII, JMJD2a and JMJD3 and reduced HDAC1/2/4, Setdb1 and EZH2 and DNA methylation due to the activity of DNMT1 and DNMT3A. Higher 5hmC levels were also detected on the Osterix promoter when 5mC was reduced. Overall, there was an observed reduction in histone H3 levels indicative of chromatin remodelling during osteogenesis. Chromatin remodelers, Brg1 and Brm, were found at high levels on the Osterix promoter in MSC and osteoblasts but not in non-MSC cells, suggesting chromatin remodelling allows transcription to occur in permissive cells. Of note, both Tet1 and Tet2 levels were also enhanced during osteogenesis and were associated with increased binding to the Osterix promoter. Moreover, knockdown of Tet1 and Tet2 revealed elevated H3 on the promoter, while Brg1, Brm, Jmjd2a, H3Ac and H3K4me3 levels were reduced, and repressive marks such as H3K9me3 and H3K27me3 were enhanced. The binding of COMPASS complex, WDR5 and SETD1, MLL2 and MLL4, was also dependent on Tet1 and 2 indicating that the Tet molecules act as scaffolding proteins recruiting epigenetic modulators to the promoter sites to facilitate BMSC lineage determination [29].

## Genome-Wide Epigenetic Changes During MSC Lineage Determination

Identifying genome-wide epigenetic signature changes and chromatin remodelling regions during BMSC multi-differentiation enables the identification of essential genes, promoters and enhancers that function in lineage determination. Studies of histone modifications have been examined using C3H10T1/2 cells and BMSC following osteogenic differentiation in vitro [67]. Interestingly, these studies reported that global histone modification levels remained largely unchanged. Examination of osteogenic genes by ChIP analysis upstream of the transcription start site, promoter and exon regions found Runx2 to exhibit no observable changes in occupancy of histones. However, Osterix, alkaline phosphatase, bone sialoprotein and osteocalcin all showed decreases in histone 3 upstream of and including the promoters [67]. Furthermore, H3K9 and H3K14 acetylation increased upstream of the transcription start site and promoter region for all genes. Whilst no differences were seen in H3K4me1 and H3K4me3 levels, H3K4me2 increased upstream of transcription start site for Runx2 and Osterix. In contrast, H3K9me2 and H3K27me3 levels decreased for both Runx2 and Osterix. When examining BMSC derived from osteoporotic samples, there were low levels of H3K9 and H3K14 acetylation and H3K4me2, and higher levels of H3K27me3 on Runx2 and Osterix promoters and upstream regions correlating with reduced transcription.

Whole-genome histone modifications and DNA methylation have been mapped during chondrogenic differentiation of human BMSC [68]. The transcriptional permissive marks H3K4me3, H3K9Ac and H3K36me3 showed similarity between donors. While H3K4me3 and H3K9Ac levels were upregulated in activated genes, H3K36me3 showed the strongest correlation, whereas H3K27me3 was increased in repressed genes. H3K27me3 appeared to decrease in chondrogenic associated genes. Downregulated genes showed a decrease in H3K4me3, H3K9Ac and H3K36me3, with the majority of activated genes showing increased H3K4me3 levels. Gene ontology analysis of H3K4me3 genes showed enrichment for skeletal development, extracellular matrix molecules and chondrocyte differentiation. Genes with downregulated H3K4me3 were enriched for apoptosis, development and metabolism pathways. Genes that were not upregulated but had H3K4me3 did not exhibit increased levels of H3K9Ac or H3K36me3. Based on the enhancer marks H3K4me1 and H3K27Ac, the majority of upregulated genes were in the vicinity of enhancers. Chondrogenic signature genes were found to be hypomethylated before and after differentiation illustrating that DNA methylation has no significant role in chondrogenesis. A similar study examining chondrogenesis in vitro employed a gene stratification analysis into low, middle and high expression. Histone methylation marks H3K4me3, H3K27Ac, H3K4me1 and H3K36me3 were associated with high expressing genes [69]. Conversely, H3K27me3 was associated with low expressing genes. In differentiated chondrocytes, the H3K4me1 and H3K27Ac enhancer marks correlated with chondrogenesis and cartilage function. When compared to the NIH road map, which entails chromatin states of over a hundred different cell types, there was no significant clustering when excluding enhancer marks. When enhancer marks were examined, they clustered with similar cell types such as chondrocytes derived from BMSC. In addition, the Human Methylation 450k bead ChIP array was used to examine DNA methylation showing demethylation predominantly on genes related to chondrogenesis. This is in contrast to other studies that showed limited DNA methylation changes, which were biased towards promoters.

Genome-wide epigenetic map analysis during adipogenesis, using human adipose-derived MSC and embryonic derived 3T3L1 cells, found increased H3K4me3 levels along with gene promoters during adipogenesis, while H3K4me1 and H3K27Ac marks were found mainly in promoters, introns and intergenic regions [70]. H3K36me3 levels were found across transcribed bodies that increased during adipogenesis. The repressive mark, H3K27me3, was distributed broadly associated with inactive genes. Moreover, H3K4me1 and H3K27Ac levels changed dramatically during differentiation in adipogenic regulated genes, correlating to increased adipocytes, and H3K27Ac levels in all regions. In a study examining the adipogenic/osteogenic potential of murine BMSC, the ability of the cells to transdifferentiate from adipocytes to osteoblasts was evident at the epigenetic level, implying a plasticity property following lineage maturation [71].

Gene ontology analyses investigating changes in epigenetic profiles of murine BMSC during osteogenic differentiation revealed genes associated with cell cycle, DNA replication and non-bone lineage transcription factors were downregulated, whereas bone differentiation genes were upregulated [72]. Highly upregulated genes showed some changes in H3K4me3 but more so with H3K9Ac. The H3K27Ac and H3K36me3 marks showed a closer relationship, which increased for upregulated genes and decreased for downregulated genes. In addition, most upregulated genes showed a decrease in H3K27me3 levels, whereas downregulated genes exhibited no overall changes in H3K27me3 levels. Genome-wide DNA methylation profiles during osteogenic differentiation of human BMSC indicate that the UTR region of genes showed no change in methylation whereas the promoter, exon, intron and intergenic regions showed significant changes [73]. Binding sites for transcription factors involved in maintaining the immature phenotype were hypermethylated, in contrast to differentiation inducing transcription factors, which were largely hypomethylated. Therefore, initiation of MSC commitment requires a co-ordinated suppression of genes associated with MSC stemness, whilst at the same time allowing the opening of chromatin containing differentiation-associated genes for transcription to occur. This process appears to be dependent on epigenetic modification patterns at specific sites along with whole genes.

## Therapeutic Targeting of Epigenetic Regulators in Osteoporosis

The use of EZH2 inhibitor GSKJ126 reduces H3K27me3 around transcription start sites, enhancing expression of osteogenic initiating factors, PTH, BMPs, Wnt genes and extracellular matrix genes in murine MC3T3 cells [74••], resulting in enhanced mineral formation. Micro-computed tomography and histomorphological analyses showed that GSKJ126 treatment of mice increased cortical bone thickness in 2-month-old femora as well as osteoblast numbers. This was also seen in an ovariectomy mouse model as GSKJ126 mitigated bone loss, increased cortical and trabecular bone in femurs of ovariectomised mice. Furthermore, studies of BMSC isolated from bone samples of osteoporotic mice following bilateral ovariectomy demonstrated that EZH2 was overexpressed in BMSC resulting in increased levels of the repressive mark H3K27me3 on Wnt1, 6 and 10a [21]. Treating osteoporotic bone-derived MSC with the EZH2 chemical inhibitor, DZnep, increased Wnt expression and enhanced osteogenesis whilst reducing adipogenesis [21].

An osteoporotic model based on injecting dexamethasone into the tibia of mice showed an increase in adipogenesis and expression of adipogenic genes, PPARγ2, C/EBPα and GLUT4, and a reduction in osteogenic genes Runx2, alkaline phosphatase, bone sialoprotein and Osteocalcin [75]. Increased DNA demethylation was observed on the PPARγ2 promoter along with increases in H3K9, H3K14 and H3K12 acetylation in MSC derived from osteoporotic bone samples. H3K9me2 levels also decreased corresponding to a decrease in HDAC1, SETDB1 and an increase in the H3K9 demethylase, LSD1. Other studies examining induced bone loss in the femora of rats, due to the lack of mechanical loading, reported a decrease in the long coding RNA H19 coinciding with increased CpG methylation along with its promoter [76] due to an increase in DNMT1 activity. H19 activates the Wnt and ERK pathways hence its decrease leads to decreased osteogenesis. Studies of siRNA mediated knockdown of DNMT1 gene expression in the femora of rats were also found to enhance osteogenesis and trabecular number and bone volume.

The H3K36me3 demethylase, SETD2, has been shown to be downregulated during adipogenesis, whereas SETD2 levels increase during osteogenesis [51]. Analysis of SETD2 deficiency in the mesenchymal lineage showed enhanced adipogenesis and reduced osteogenesis, where SETD2 knockout mice exhibited reduced cortical bone, trabecular bone number and bone volume, akin to an osteoporotic-like phenotype. Purified BMSC from these mice showed differential changes in gene expression patterns, correlating to a dramatic reduction in H3K36me3 levels mainly occurring in gene bodies, promoters, 3′UTR, and intergenic regions [51]. Furthermore, SETD2 was found to be decreased in aged murine BMSC (20–60 weeks), supporting other findings that H3K36me3 promotes longevity [51].

The H3K4 methylation mark is associated with gene activation, where the H3K4 demethylase, KDM5A, was found to be specifically upregulated in both human and mouse BMSC derived from osteoporotic bone samples [31]. Functional studies demonstrated that overexpression of KDM5A causes inhibition of BMP-2 induced BMSC osteogenesis. ChIP analysis of chromatin isolated from KDM5A overexpressing BMSC found lower levels of H3K4me3 on the Runx2 promoter. The KDM5A mediated inhibition of BMSC osteogenic differentiation was restored by the addition of specific KDM5A short hairpin RNA or inhibitor. Moreover, bone loss in osteoporotic mice, was partly rescued following pre-treatment with a chemical inhibitor to KDM5A activity, demonstrating the regulatory role of KDM5A in osteoporosis in mice [31].

**Fig. 3:**
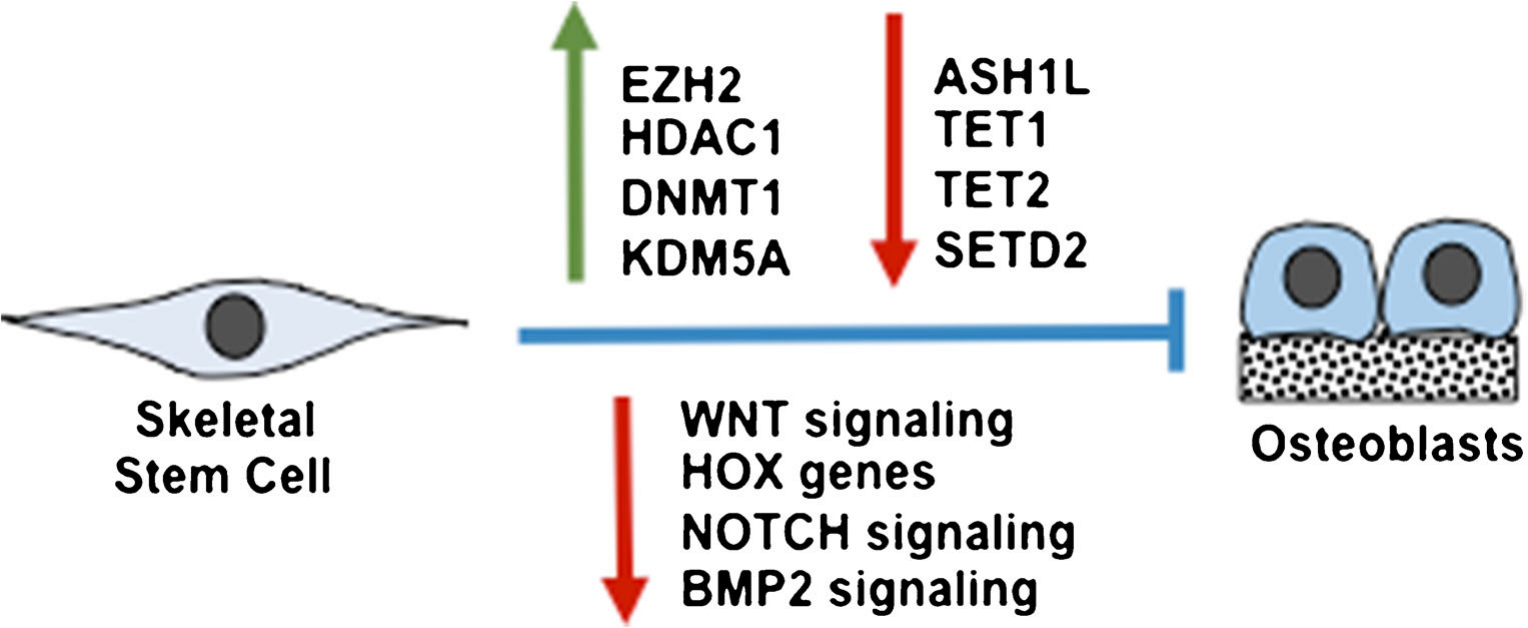
Epigenetic enzymes deregulated during osteoporosis. Epigenetic enzymes deregulated in skeletal stem cells during the onset of osteoporosis leading to reduced osteoblast numbers and function. Upregulation of EZH2 and upregulation of DNMT1 inhibits WNT signalling; downregulation of ASH1L l suppresses HOX genes; upregulation of HDAC1 inhibits Notch signalling; downregulation of TET1/2 suppresses Runx2; downregulation of SETD2 and upregulation of KDM5A inhibits BMP2 signalling

Collectively, these preclinical studies highlight the reversible nature of the epigenetic landscape under pathological settings, which is amenable to drug targeting (Fig. 3). However, it remains to be determined whether chemical inhibitors targeting epigenetic factors are indeed specific to the appropriate enzymes, where many of these are currently being assessed in human cancer trials. The issue of targeting skeletal tissue using bone-specific carrier vehicles is also currently under investigation using various approaches. Finally, the activity of epigenetic inhibitors to prevent bone loss in diseases such as osteoporosis must be considered with caution due to unexpected consequences such as the potential to diminish the function or pool of skeletal stem cells over time.

## Conclusions

Epigenetic regulation of MSC differentiation is an area that has progressed rapidly in the last decade with the use of technologies such as ATAC-seq, ChIP seq, bisulphite sequencing and 3C-chromatin capture. With more than 20 known histone modifications and two key DNA modifications, the potential for unique epigenetic combinations is staggering, illustrating the field is still in its infancy. The literature up to now shows that during differentiation, activating histone modifications consisting of combinations of H3K4me1/2/3, H3K36me3, H3R17, H3K9Ac and H3K27Ac prevail along with lineage-specific genes related to osteogenesis, adipogenesis and chondrogenesis, depending on the differentiation path. The most tightly associated mark with lineage transcription is H3K36me3/H3K4me3 along with gene bodies and transcription start sites together with H3K4me1 and H3K27Ac that mark enhancers. The enhancer modifications closely correlate with active lineage-associated transcription and are sites of active chromatin remodelling. Commonly seen is the appearance of repressive marks (H3K27me2/3, H3K9me2) on lineages not activated to ensure their repression. Although DNA methylation on promoters is not entirely consistent with BMSC differentiation, DNA methylation along with gene bodies and enhancers is closely linked with transcription as removal of DNA methylation marks is associated with activation. Epigenetic deregulation in osteoporotic BMSC implies loss of cell identity with representation of non-bone markers, compensatory mechanisms cause an increase in early osteogenic gene expression to synthesise more bone and changes in the active and repressive histone marks and DNA methylation on lineage-associated genes lead to decreased osteogenesis and enhanced adipogenesis. Further investigations into the identification of novel epigenetic factors and target genes regulating MSC differentiation will help progress the development of epigenetic drug-specific targeting of skeletal tissues for a range of orthopaedic-related indications, by coupling with new drug screening and drug delivery approaches.
